# Inhibitory Effect of *Centella asiatica* Extract on DNCB-Induced Atopic Dermatitis in HaCaT Cells and BALB/c Mice

**DOI:** 10.3390/nu12020411

**Published:** 2020-02-05

**Authors:** Yonghyeon Lee, Hyeon Kyeong Choi, Kaudjhis Patrick Ulrich N’deh, Young-Jin Choi, Meiqi Fan, Eun-kyung Kim, Kang-Hyun Chung, Jeung Hee An

**Affiliations:** 1 Department of Food and Nutrition, KC University, Seoul 07661, Korea; lyh_0904@naver.com (Y.L.); cchk0726@naver.com (H.K.C.); kaudjhispatrick@gmail.com (K.P.U.N.); 2 Department of Food Science and Technology, Seoul National University of Science and Technology, Seoul 01811, Korea; carl@seoultech.ac.kr; 3 Division of Food Bioscience, College of Biomedical and Health Sciences, Konkuk University, Seoul 27478, Korea; choijang11@kku.ac.kr (Y.-J.C.); fanmeiqi@kku.ac.kr (M.F.); eunkyungkim@kku.ac.kr (E.-k.K.)

**Keywords:** atopic dermatitis, *Centella asiatica* ethanol extract, madecassoside, asiaticoside, anti-inflammation

## Abstract

Atopic dermatitis (AD) is a chronic inflammatory skin disease caused mainly by immune dysregulation. This study explored the anti-inflammatory and immunomodulatory effects of the *Centella asiatica* ethanol extract (CA) on an AD-like dermal disorder. Treatment with CA inhibited the expression of interleukin-6 (IL-6) and tumor necrosis factor-α (TNF-α) in a dose-dependent manner in inflammatory stimulated HaCaT cells by interferon-γ (IFN-γ) and TNF-α-triggered inflammation. Eight-week-old BALB/c mice treated with 2,4-dinitrochlorobenzene (DNCB) were used as a mouse model of AD. In AD induce model, we had two types treatment of CA; skin local administration (80 µg/cm^2^, AD+CA-80) and oral administration (200 mg/kg/d, AD+CA-200). Interestingly, the CA-treated groups exhibited considerably decreased mast cell infiltration in the ear tissue. In addition, the expression of IL-6 in mast cells, as well as the expression of various pathogenic cytokines, such as TNF-α, IL-4, IL-5, IL-6, IL-10, IL-17, iNOS, COX-2, and CXCL9, was reduced in both AD+CA-80 and AD+CA-200 groups. Collectively, our data demonstrate the pharmacological role and signaling mechanism of CA in the regulation of allergic inflammation of the skin, which supports our hypothesis that CA could potentially be developed as a therapeutic agent for AD.

## 1. Introduction

Atopic dermatitis (AD, also known as atopic eczema) is a highly pruritic and chronic inflammatory skin disease caused by abnormal immune responses [1] which is characterized by skin barrier dysfunction. AD is influenced by multiple immune system alterations and a variety of environmental factors (e.g., mite dust, smoking, exposure to allergens, etc.), which lead to eczematous and itchy skin lesions [2]. The disease impacts roughly 15–20% of children and 1–3% of adults ecumenical while its prevalence continues to increase, especially in low-income countries [3,4]. Recent studies have broadened the knowledge of immunological and molecular mechanisms involved in AD disease. For example, it is now known that infiltration of immune cells (e.g., T cells, mast cells, and dendritic cell subtypes) is increased in AD lesions, the serum IgE level is elevated in AD patients compared to that in patients affected by several inhaled allergens, and the increase of secreted Th2 helper cytokines is highly correlated with the disease [2,5,6]. The current common therapy is the anti-inflammatory treatment of visible skin lesions using steroids, including topical corticosteroids (e.g., glucocorticosteroids), topical calcineurin inhibitors (e.g., tacrolimus and pimecrolimus), or both [7]. Although these topical treatments are able to alleviate AD symptoms, reduce inflammation, and prevent flares, they are associated with long-term use side effects. These include local cutaneous atrophy, striae formation caused by topical corticosteroids, and stinging upon application of topical calcineurin inhibitors [8]. Thus, there is a large unmet need for safe and effective AD therapeutics. Recently, there has been a growing appeal for alternative therapeutic agents for AD treatment, especially natural bioactive compounds from plants extracts [9].

*Centella asiatica* is a medicinal plant belonging to the Apiaceae family commonly used as a traditional herbal medicine and major ingredient in nutraceutical products in Southeast Asian countries [10,11]. Moreover, *Centella asiatica* is rich in the flavonoid quercetin, which has a therapeutic effect in the context of AD- induced by 2,4-dinitrochlorobenzene (DNCB) [12,13]. The European Medicines Agency reported that no significant problems arose from either the topical application or the oral administration of *Centella asiatica* ethanol extract (CA) [14]. *Centella asiatica* is a pentacyclic triterpene-rich medicinal herb. The medicinal efficacy of the plant is mainly attributed to the most prominent bioactive triterpenes named madecassoside (MO), asiaticoside (AO), madecassic acid (MA), and asiatic acid (AA) [11]. The distribution of pentacyclic triterpenes accumulated in the plant varies according to plant parts, cultivation zone, and harvesting period [15]. *Centella asiatica* and its triterpenes have been reported to exhibit wound healing and memory improvement properties, and improve the treatment of asthma, psoriasis, ulcer, and cancer [11]. In addition, *Centella asiatica* and its active triterpenes constituents have been proved to act as an anti-allergic, anti-inflammatory, antifibrotic, cardioprotective, neuroprotective, antioxidant, antidepressant, anticancer, antibacterial, and antifungal agent [5,10,16,17,18,19]. Especially, the anti-inflammatory properties of AA were highlighted using in vitro and in vivo studies [20,21,22]. In fact, the LPS-induced inflammatory response (e.g., increased level of prostaglandin E2, nitric oxide, interleukin (IL)-6 and IL-8, and phosphorylation of p65 nuclear factor kappa B (NF-κB) in human gingival fibroblasts was inhibited by AA treatment [20]. In addition, AA has been shown to produce inhibited pulmonary inflammation induced by cigarette smoke in mice [21]. Further studies show that mice pre-treatment with AA inhibited bleomycin-induced lung injury and fibrosis progression [22]. However, there is no previous research investigating the therapeutic effects of the CA and its triterpene MO and AO in the DNCB-induced atopic dermatitis model.

We investigated the anti-inflammatory and immunomodulation effects and action-related mechanism of *Centella asiatica* leaf extracts both in vitro and in vivo. Our study elucidated in AD mouse model, the effect of CA on the ear thickness and immune cell infiltration into the dermis and epidermis, as well as the cytokine and mitogen-activated protein kinase (MAPK) levels in the ear tissue. Additionally, we evaluated the *Centella asiatica* leaf extract in vitro anti-inflammatory and in vivo anti-dermatitis effects, as well as the bioactive triterpene aglycone AA effect on the DNCB-induced atopic dermatitis in the mouse model.

## 2. Materials and Methods

### 2.1. Plant Material and Extraction

Dried leaves of Korean GOOD *Centella asiatica* were obtained from Centella Farm (Chungju, Korea). The CA was obtained by mixing ethanol with 300 g of the *Centella asiatica* in a 10-fold (*v*/*w*) dilution, shaking the solution at 25 °C for 24 h, and performing filtration through Whatman #2 filter paper. The filtered extract was concentrated using a rotary vacuum concentrator (R-114, Buchi Co., Flawil, Switzerland), and the solvent was dried using a vacuum concentrator (Ecospin 314, PS1E1AF01, Bio-tron Inc., Bucheon, Korea) for three days. The ethanol extract (45.0 g, 15% extract yield) was stored at −80 °C until use.

### 2.2. High Performance Liquid Chromatography (HPLC) Analysis

The ethanol extract was accurately weighed (100 mg), dissolved in methanol:water solution (90:10), and filled up to 10 mL. The solution was filtered with a 0.45-µm filter and used in high-performance liquid chromatography (HPLC) analysis. A reverse-phase column (SunFire C18, 4.6 × 250 mm, 5-µm diameter; Waters, Milford, MA, USA) and the HPLC empower software (Waters, Milford, MA, USA) were used to analyze the compounds present in the extract. A 10 µL sample volume was injected onto the column and the chromatograms were detected at 206 nm. MO, AO, and AA were purchased from Sigma-Aldrich (St. Louis, MO, USA), and MA was purchased from Abcam (Cambridge, UK), to be used as authentic standards.

### 2.3. Determination of Total Polyphenols, Flavonoid, and Antioxidant Capacities of Centella asiatica Extract (CA)

The total polyphenol content of CA was determined by modifying the method of Folin-Denis [23]. A standard calibration curve was plotted using gallic acid (5–1000 mg/L) (Sigma-Aldrich Co., St. Louis, MO, USA). The results were expressed as mg of gallic acid equivalents (GAE)/100 g of extract.

The total flavonoid content was measured using the method of Zhishen et al. (1999) [24]. A standard calibration curve was plotted using (+)-catechin hydrate (Sigma-Aldrich Co.). The results were expressed as mg of (+)-catechin hydrate equivalents (CHE, dry basis)/100 g of extract.

The 2,2′-azinobis-(3-ethylbenzothiazoline-6-sulfonic acid) (ABTS) method was based on the method outlined by Re et al. [25]. The absorbance was determined at 734 nm with a UVM 340 microplate reader (Biochrom Asys, Cambridge, UK). The resulting value was expressed as a percentage (%) of the radical scavenging activity by comparing the extract addition group and the control group.

The 2,2-diphenyl-1-picrylhydrazyl (DPPH) radical scavenging activity of CA was measured according to the method of Blois et al. [26]. The absorbance was determined at 517 nm using the UVM 340 microplate reader. The resulting value was expressed as a percentage (%) of the radical scavenging activity by comparing the extract addition group and the control group.

### 2.4. Cell Culture and Stimulation

HaCaT cells (Korean Cell Bank, Seoul, Korea) were cultured in Dulbecco’s modified Eagle’s medium containing 10% fetal bovine serum (Hyclone, Logan, UT, USA) and 1% penicillin-streptomycin antibiotic (GIBCO, Grand Island, NY, USA) in a humidified incubator at 37 °C under 5% CO2. The HaCaT cells were incubated for 24 h at 1 × 10^5^ cells/mL. After the cells had been pretreated with CA (100 µg/mL, 300 µg/mL, and 500 µg/mL) for 1 h, they were stimulated for 24 h with interferon-γ (IFN-γ; 10 ng/mL) and tumor necrosis factor-α (TNF-α; 10 ng/mL) as inducers of inflammation for 24 h.

### 2.5. Cell Viability

We cultured HaCaT cells in a 96-well plate, and cell viability was analyzed using the 3-(4,5-dimethylthiazol-2-yl)-2,5-diphenyltetrazolium bromide (MTT) (Promega, Madison, WI, USA) assay. Briefly, the cells were treated with various concentrations of CA, MO, and AO (CA: 10–1000 µg/mL; MO: 10–300 µg/mL; AO: 10–300 µg/mL). The MTT solution was then added, followed by incubation at 37 °C for 4 h. The supernatant was removed, and the formed formazan crystals were dissolved in dimethyl sulfoxide. The absorbance was measured at 540 nm by the UVM 340 microplate reader.

### 2.6. Animals and Treatment

**Figure 1:**
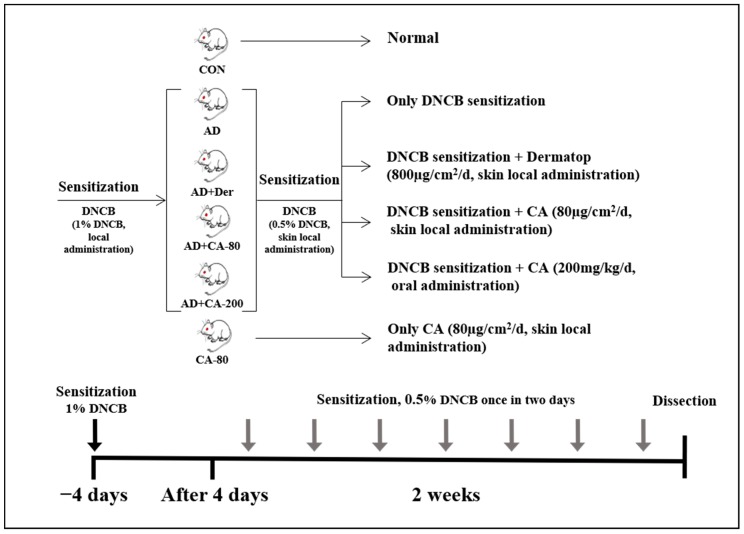
Experimental design of atopic dermatitis (AD) -like skin lesions mice model. BALB/c mice were randomly divided into six groups (*n* = 8 per group). AD-like skin lesions were evoked by administering 1% 2,4-dinitrochlorobenzene (DNCB). 200 µL of 0.5% DNCB was applied on each ear, and after 4 days. DNCB was alternatively repeatedly treated once in two days for 2 weeks. *Centella asiatica* ethanol extract (CA) was administrated by skin local administration (80 µg/cm^2^/d) and oral administration (200 mg/kg/d). The Dermatop was skin local administrated (800 µg/cm^2^/d). Without inducing AD, CA (80 µg/cm^2^/d) was skin locally administrated for 2 weeks.

Eight-week-old female BALB/c mice were purchased from Samtako (Osan, Korea) and housed under specific pathogen-free conditions. The mice were housed in transparent plastic cages bedded with aspen chips and provided with a standard mouse diet and tap water ad libitum when not being treated. The environment of the animal room was carefully controlled, with a 12 h dark–light cycle, temperature of 20–21 °C, and relative humidity of 40–45%. All experiments were approved by the institutional animal care and use committee of Konkuk University (KU19107) and were performed in accordance with relevant guidelines and regulations. In our study, BALB/c mice were randomly divided into two main groups (*n* = 8 per group) composed of the AD treatment groups and non-AD treatment groups. AD treatment groups included AD group (0.5% DNCB-sensitized in 200 µL of acetone/bean oil-3:1), AD+CA-80 group (80 µg/cm^2^ CA in 200 µL of sterilized drinking water), AD+CA-200 group (200 mg/kg CA in 200 µL of sterilized drinking water), and the positive control named AD+Der group (800 µg/cm^2^ dermatop containing prednicarbate at 0.25% *w*/*w*, SANOFI-ADVENTIS KOREAM CO., Korea) [27,28]. Non-AD groups were divided into CON group (control group) and CA-80 group (CA 80 µg/cm2): (Figure 1). In the groups with induced AD, repeated local exposure of the ears to DNCB was performed, as previously described [29]. Ear thickness was measured 24 h after DNCB application with a dial thickness gauge (Kori Seiki MFG Ltd., Tokyo, Japan). On the 14th day, mice ears were excised and subjected to histopathological analysis. The weight of lymph nodes was measured with an CP-224S electronic balance (Sartorius, Göttingen, Germany).

### 2.7. Histological Observations and Immunohistochemical Staining (IHC)

Mice ears were fixed with 10% formaldehyde for 24 h, embedded in paraffin, and cut into 4 µm-thick slices. The sections were stained with hematoxylin and eosin staining for routine histopathological examination and were evaluated using a light microscope (Eclipse TE 200; Nikon, Tokyo, Japan) at 100× magnification. For the measurement of mast cell infiltration, ear sections were stained with toluidine blue, after which the number of mast cells was counted in three randomly chosen fields of view at 400× magnification. Ear sections (4 µm-thick) were incubated with 10% goat serum (for polyclonal antibodies) for 30 min, and then incubated at 4 °C with a primary antibody against NF-κB (Abcam, Cambridge, UK) for 24 h. Subsequently, all samples were incubated with biotinylated goat anti-rabbit immunoglobulin G (H+L) horseradish peroxidase-conjugated antibodies (Zymax, San Francisco, CA, USA). The specimens were examined using a Nikon Eclipse TE 200 (Nikon) at 200× magnification, and the microscopy images were analyzed using the OptiView image analysis software (Korea LabTech Corp., Seongnam, Korea).

### 2.8. Real-Time PCR

The total RNA was isolated from mice ear tissue using TRIzol (Invitrogen Co., Carlsbad, CA, USA) according to the manufacturer instructions [30]. The first-strand complementary DNA (cDNA) was synthesized using master mix kit for cDNA synthesis (Bioneer Co., Daejeon, Korea). Real-time PCR was performed in triplicate using 12.5 µL of SYBR Premix Ex Taq (Takara, Tokyo, Japan) and 2 µL of cDNA as a template in 25 µL of final volume. PCR amplification was preceded by mixture incubation for 15 min at 95 °C, and 40 cycles of the amplification step. The denaturation phase was performed for 30 s at 95 °C; annealing was performed in a transitional temperature range from 58 to 62 °C, with an increase of 0.5 °C per cycle; and the extension was performed for 30 s at 72 °C with fluorescent detection at 72 °C after each cycle. After the final cycle, melting point analyses of all samples were performed within the range from 65 to 95 °C with continuous fluorescent detection. Target gene mRNA levels were normalized to glyceraldehyde 3-phosphate dehydrogenase (GAPDH) levels using the following formula: relative mRNA expression =$2^{–\left(\Delta \mathrm{Ct}\;\mathrm{of}\;\mathrm{target}\;\mathrm{gene}-\Delta \mathrm{Ct}\;\mathrm{of}\;\mathrm{GAPDH}\;\mathrm{gene}\right)}$, where *Ct* is the threshold cycle value. In each sample, the expression level of the analyzed gene was normalized to that of GAPDH and presented as a relative mRNA level. The PCR primers used are listed in the Supplementary Material (Table S1).

### 2.9. Western Blotting

Cells and mouse ears were homogenized in lysis buffer containing protease inhibitors (Roche, Mannheim, Germany) and were then centrifuged at 4 °C, 10,000× *g* for 30 min. Total soluble protein contents were evaluated using a Bio-Rad protein kit (Bio-Rad, Laboratories, Hercules, CA, USA). The proteins were separated by electrophoresis and transferred onto immobilon-P transfer membranes (Millipore, Burlington, MA, USA). The membranes were blocked with 5% bovine serum albumin. Subsequently, the membranes were incubated at 4 °C for 24 h with specific primary antibodies against p-p38, phosphorylated extracellular signal-regulated kinase (p-ERK) and β-actin (Cell Signaling Technology, Beverly, MA, USA), TNF-α, NF-κB, inducible Nitric Oxide Synthase (iNOS), Cyclooxygenase-2 (COX-2) (Abcam), IL-6 (Santa Cruz Biotechnology, Santa Cruz, CA, USA), and macrophage-1 (MAC-1) (Bio-Rad). The membranes were incubated with a goat anti-rabbit immunoglobulin G (H+L) horseradish peroxidase-conjugated secondary antibody (Zymax). Protein bands were visualized by enhanced chemiluminescence and densitometric analysis of the protein bands were performed by a C-DiGit Blot Scanner (Li-COR, Lincoln, NE, USA) and ImageJ (NIH, Rockville, MD, USA). All data were normalized to the β-actin values.

### 2.10. Statistical Analyses

All statistical analyses were performed using SPSS version 18.0 (IBM, Chicago, IL, USA). One-way analyses of variance with Duncan’s post hoc tests were used to identify differences in the mean values between experimental groups. Data for each test are presented as mean ± standard deviations. Statistical significance was set at *p* < 0.05.

## 3. Results

### 3.1. HPLC Analysis

**Figure 2:**
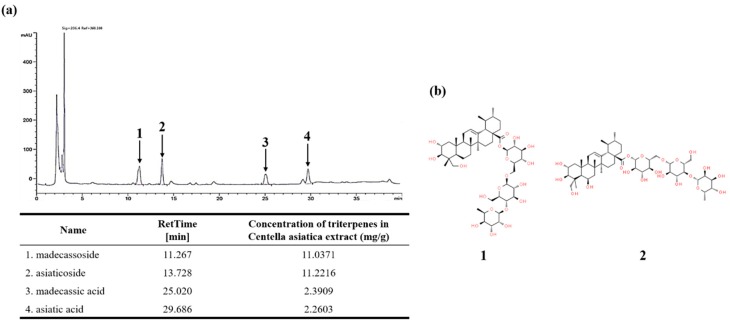
(**a**) High-performance liquid chromatography (HPLC) chromatography of CA; (**b**) Chemical structures of major components. The HPLC analysis on CA was performed with a reverse-phase column (SunFire C18, 4.6 × 250 mm, 5-um diameter; Waters, Milford, MA, USA). The column was preserved under 40 °C at the flow rate of 1 mL/min and the injection volume of 10 µL. 1: madecassoside (MO), 2: asiaticoside (AO), 3: madecassic acid (MA), 4: asiatic acid (AA).

The medicinal values of *Centella asiatica* are mainly attributed to the presence of four triterpenes, namely MO, AO, MA, and AA. Using these components as the standard, the content of the corresponding components of CA were analyzed using HPLC. AO of CA is the most abundant component, followed by MO at 11.22 mg/g and 11.03 mg/g, respectively. Both MA and AA were found at 2.39 mg/g and 2.26 mg/g, respectively. All components were detected in CA, and MO and AO were extracted with an approximate 5-fold increase compared to MA and AA (Figure 2a). Thus, our HPLC analysis showed that the major components in CA are MO and AO.

### 3.2. Total Polyphenols and Flavonoid Content and Free Radical Scavenging Activity of CA

**Figure 3:**
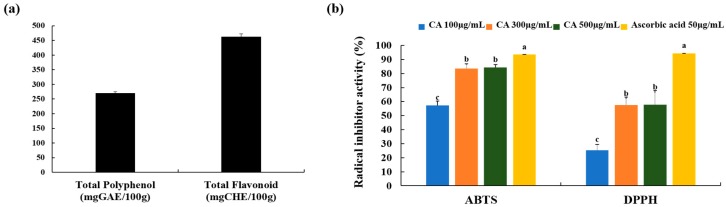
Polyphenol and flavonoid content, and antioxidant capacities of CA. (**a**) Total polyphenol and flavonoid; (**b**) 2,2’-azinobis-(3-ethylbenzothiazoline-6-sulfonic acid) (ABTS) and 2,2-diphenyl-1-picrylhydrazyl (DPPH) scavenging activity. Values represent the mean ± SD. Values with different letters were significantly different according to Duncan’s multiple range test (*p*< 0.05). GAE: gallic acid equivalents, CHE: (+)-catechin hydrate equivalents.

The total phenolics of CA were evaluated to be 269.76 mg GAE/100 g of *Centella asiatica*, using a standard curve of gallic acid (*R*^2^ = 0.9959). The total flavonoid contents of CA were evaluated to be 462.42 mg CHE/100 g of *Centella asiatica*, using a standard curve of (+)-catechin hydrate (*R*^2^ = 0.9965) (Figure 3a).

We examined the antioxidant activity of CA by performing an ABTS and DPPH radical scavenging activity assay (Figure 3b). The ABTS and DPPH radical scavenging activity of CA (100 µg/mL and 300 µg/mL) was concentration-dependent, but it was no statistical differences between the 300 µg/mL and 500 µg/mL of CA (Figure 3b). Ascrobic acid (50 µg/mL) was used as positive control. The ABTS radical scavenging activity dramatically increased in 300 µg/mL and 500 µg/mL of treated CA (Figure 3b). Furthermore, the DPPH radical scavenging activity in the 300 µg/mL and 500 µg/mL CA-treated groups (32.12% and 32.35%, respectively) were enhanced compared to a 100 µg/mL of CA concentration (Figure 3b). The results suggest potent antioxidant activity is justified by the contents of polyphenol, flavonoid and four triterpenes: MO, AO, MA, and AA [11,31].

### 3.3. CA Attenuated Inflammatory Stress in Stimulated Keratinocytes

**Figure 4:**
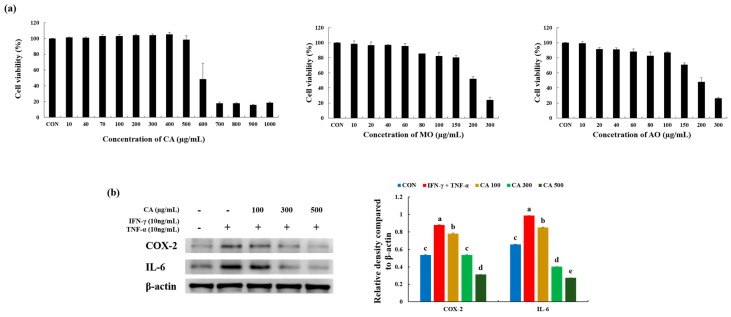
(**a**) 3-(4,5-dimethylthiazol-2-yl)-2,5-diphenyltetrazolium bromide (MTT) assay for the cell toxicity of CA, MO, and AO in HaCaT cells treated with different concentrations of CA at 37 °C for 24 h under 5% CO_2_; (**b**) Effects of CA on the expression of inflammation-related proteins in HaCaT cells stimulated by tumor necrosis factor-α (TNF-α) and interferon-γ (IFN-γ) in a CA dose-dependent manner. Values represent the mean ± SD. Values with different letters were significantly different according to Duncan’s multiple range test (*p* < 0.05).

To determine the cell toxicity of CA, HaCaT cells were treated with CA in a dose-dependent manner (Figure 4a). No cell toxicity was assessed using a CA concentration in the range of 10 µg/mL to 500 µg/mL. With an increased CA concentration from 600 µg/mL, cell viability drastically decreased to 48.46%. In addition, no cytotoxic effects were observed in this cell line at 10–150 µg/mL concentrations of both MO and AO (Figure 4b).

The effects of CA on HaCaT keratinocytes co-stimulated by pro-inflammatory mediators, IFN-γ (10 ng/mL) and TNF-α (10 ng/mL), were assessed using a western blot analysis (Figure 4b). Expression of COX-2 was enhanced in treatment with IFN-γ (10 ng/mL) and TNF-α (10 ng/mL) vs. CON group (1.6-fold). This elevated expression was reduced by treatment with 100 µg/mL, 300 µg/mL, and 500 µg/mL, respectively, of CA more than the IFN-γ + TNF-α group (0.9, 0.6, 0.4-fold, respectively). The expression of IL-6 was higher in the IFN-γ (10 ng/mL) and TNF-α (10 ng/mL) groups than the CON group (1.5-fold). Increased IL-6 secretion was decreased by 0.9-, 0.4-, and 0.3-fold, respectively, in the groups treated with CA (100 µg/mL, 300 µg/mL, and 500 µg/mL) in a dose-dependent manner. Thus, CA treatment had a dose-dependent effect on inflammatory relief in HaCaT-induced inflammation by TNF-α and IFN-γ.

### 3.4. Effects of CA on Atopic Dermatitis (AD)-Like Skin Lesion in BALB/c Mice

**Figure 5:**
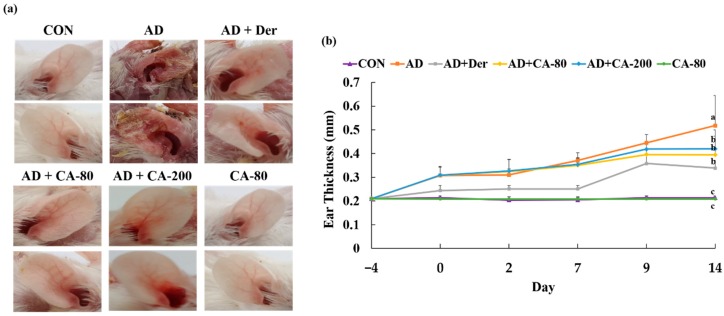
Histopathological analysis to assess the effect of CA on mice ear thickness. (**a**) Photographs of mice ears from each group on day 14; (**b**) The ear thickness was measured 24 h after DNCB application with a dial thickness gauge. Values represent the mean ± SD. Values with different letters were significantly different according to Duncan’s multiple range test (*p* < 0.05).

To investigate the effect of CA on AD, a BALB/c AD model was set up by applying DNCB on the mice earlobes for two weeks. Repeated skin local application of DNCB significantly increased ear thickness in mice (2.4-fold) compared to the CON group (*p* < 0.05) (Figure 5a,b). In addition, treated CA groups (AD+CA-80 and AD+CA-200) reduced the ear thickness (1.3- and 1.2-fold) more than the CON group. DNCB also induced remarkable AD lesions such as hemorrhage, edema, excoriation, and scaling, diminished by CA treatment (Figure 5a). Since AD often expands as a systemic immune response, it can affect the immune organs [32].

**Figure 6:**
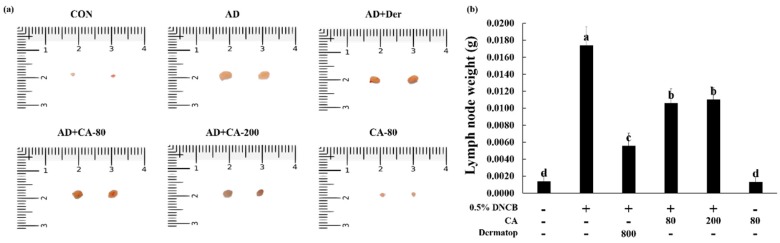
(**a**,**b**) Photographs represent the size and weight of lymph nodes. Values represent the mean ± SD. Values with different letters were significantly different according to Duncan’s multiple range test (*p* < 0.05).

Therefore, we evaluated the weight of the lymph nodes measured in the 14th day to examine whether the topical application of CA provided an anti-AD effect in mice (Figure 6a,b). Compared to the CON group, the lymph nodes of AD group increased in ear thickness (12.4-fold). However, AD+CA-80 and AD+CA-200 equally reduced the weight of mice lymph nodes (1.6-fold). The weight of lymph nodes in the CA-80 group were not significantly different from the CON group. These observations suggested that CA treatment alleviated AD in atopic dermatitis-induced mice. These observations suggested that CA contributes to the alleviation of AD-like symptoms.

### 3.5. Effects of CA on the Histopathological Aspects

**Figure 7:**
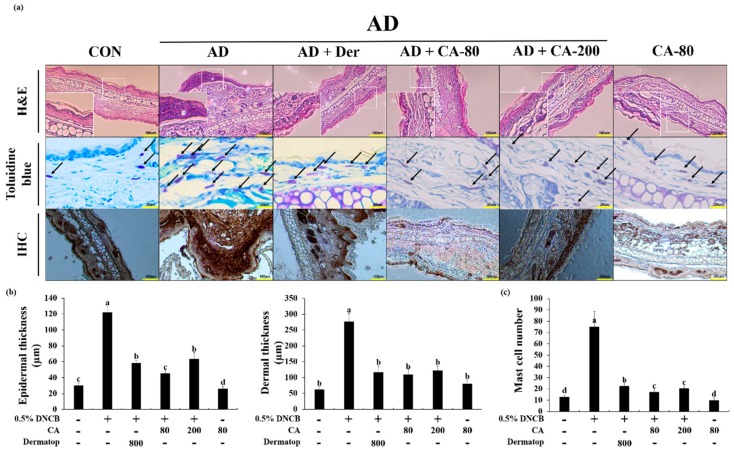
(**a**) Histological analysis to assess the effect of CA on epidermal and dermal thickness, and mast cell infiltration. Representative photomicrographs of ear sections stained with hematoxylin and eosin (H&E) or toluidine blue. In the toluidine blue staining panel, black arrows denote mast cells. In immunohistochemistry (IHC), a strong difference in staining intensity for NF-κB between atopic dermatitis model and CA treatment model was noted; (**b**) Photographs were taken under a regular light microscope at a magnification of 100× (H&E and IHC) and 400× (toluidine blue staining); (**c**) The epidermal and dermal thickness was gauged using microphotographs of hematoxylin and eosin stained tissue. The number of infiltrated mast cells was counted on the basis of toluidine blue staining. Values represent the mean ± SD. Values with different letters were significantly different according to Duncan’s multiple range test (*p*< 0.05).

To analyze the effect of CA on skin hypertrophy and granulocyte infiltration, the ear sections were stained and observed under an optical microscope. Repeated DNCB exposure caused potent inflammatory changes, such as thickening of the dermis and epidermis in the ear tissue of the AD mice (4.0- and 4.4-fold, respectively) with reference to the CON group (Figure 7a,b). More AD+CA-80 and AD+CA-200 groups significantly reduced the epidermal (2.7- and 1.9-fold) and dermal (10.1- and 5.9-fold) thickness when compared with the AD group (*p* < 0.05). Interestingly, the epidermal thickness of the AD+CA-80 group decreased (1.4-fold) at a greater scale than the AD+CA-200 group. Additionally, the epidermal thickness in the AD+CA-80 group decreased more notably (1.3-fold) than the positive control. In the CA only treated group, epidermal thickness was similar to the CON group, but dermal thickness increased 1.3-fold compared to the CON group. To further investigate these changes, we evaluated the effects of CA on the infiltration of mast cells, important effector cells, and primary source of AD histamine into the mice ears. In the AD group, mast cell number increased 5.8-fold compared to the CON group. Compared to the AD group, CA treatment groups (AD+CA-80 and AD+CA-200) reduced mast cell infiltration (4.4- and 3.7-fold, respectively) (Figure 7a,c). Additionally, the number of mast cell in AD+CA-80 group significantly reduced (1.3-fold) more than the positive control. The mast cell number of the CA group decreased in comparison to the CON group (1.3-fold). We examined the immunolocalization of NF-κB in the ear of AD mice, and in healthy controls. NF-κB was the highest detected in the AD group among all tested groups (Figure 7a). In two types of CA treatment groups, expression of NF-κB substantially decreased compared with the AD group. Thus, CA treatment decreased the thickness of epidermis and dermis, as well as the infiltration of mast cells induced by DNCB. CA also modulated the expression of NF-κB in the ears of AD-induced mouse model.

### 3.6. Effect of CA on the mRNA Expression of Th1-type, Th2-type, and IL-17 cytokines in BALB/c AD-Mice

**Figure 8:**
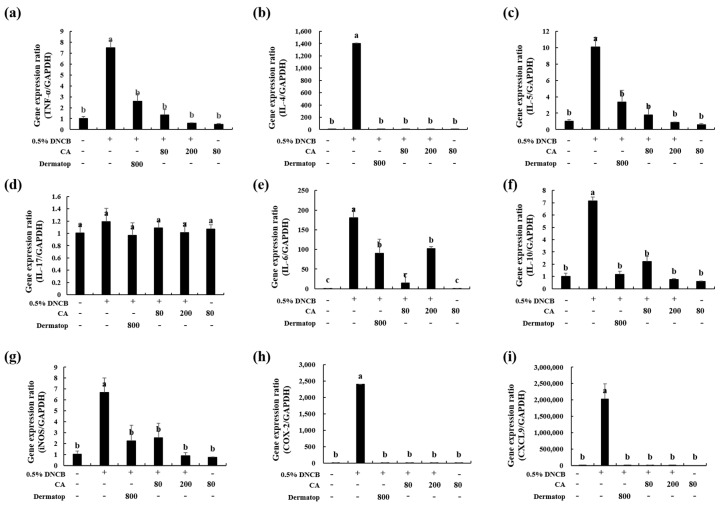
Effect of CA on the expression of diverse pathogenic factors in the ear. (**a**), TNF-α; (**b**), IL-4; (**c**), IL-5; (**d**), IL-17; (**e**), IL-6; (**f**), IL-10 (**g**), iNOS; (**h**), COX-2; (**i**), CXCL9. The total RNA was isolated from the ear tissue. Quantitative real-time PCR was performed as described in the Materials and Methods. Values represent the mean ± SD. Values with different letters were significantly different according to Duncan’s multiple range test (*p* < 0.05).

To understand the mechanism of CA in alleviating AD response, we examined the expression levels of AD-related inflammatory cytokines from the ear tissue by real-time PCR. The mRNA levels of the Th1-related cytokine TNF-α, the Th2-related cytokines IL-4, IL-5, IL-6, and IL-10, as well as the Th17-related cytokine IL-17, were examined (Figure 8a–f). The gene expression of TNF-α was upregulated in the ear tissue of the AD-treated group by 7.4-fold compared with the CON group (Figure 8a). However, in the AD+CA-80 and AD+CA-200 groups, the expression of TNF-α mRNA was downregulated by 5.6-fold and 12.9-fold, respectively, when compared with that in the AD group. The TNF-α mRNA levels in AD+CA-80 and AD+CA-200 groups showed a marked decreased (1.9- and 4.4-fold, respectively) with respect to the positive control (AD+Der). Moreover, DNCB treatment markedly raised the level of IL-5 (9.9-fold) and IL-10 (7.0-fold) in the AD group in contrast with the CON group (Figure 8c,f). As compared to the AD group, the AD+CA-80 and AD+CA-200 groups exhibited substantially inhibited the expression of IL-5 and IL-10 (reduced by 5.6- and 11.5-fold, respectively), as well as that of IL-10 mRNA level by 3.2- and 9.5-fold, respectively. In addition, the activation of IL-4 and IL-6 in the AD group significantly increased compared with the CON group (Figure 8b,e). However, CA treatment had significantly reduced the mRNA expression of IL-4 in the group only treated with AD (*p* < 0.05). The IL-6 mRNA level was considerably reduced by 12.6-fold in AD+CA-80 compared with the AD group. Similarly, the IL-6 expression was inhibited (1.7-fold) in contrast to the AD group. The expression level of IL-6 in the AD+CA-80 group decreased more notably (12.6-fold) than the positive control. The IL-17 mRNA expression level, related to Th17 cells, was not significantly different among all groups. In all the tested mRNA expression levels, results from the group only treated with CA were similar to the CON group. In AD+CA-80 and AD+CA-200, no cytokines except IL-6 were significantly different from the AD+Der group. Thus, we concluded that CA treatment reduced the mRNA expression of Th1- and Th2-type cytokines in BALB/c AD-mice.

### 3.7. Effect of CA on the mRNA Expression of Inflammatory Factors in BALB/c AD-Mice

To explain the mechanism of CA in mitigating AD response, we measured the mRNA expression of AD-related chemokines from ear skin tissue by real-time PCR (Figure 8). DNCB treatment significantly increased the activation of iNOS (6.5-fold, *p* < 0.05) in the AD group compared with the CON group. However, local skin administration of CA substantially downregulated iNOS expression in AD+CA-80 compared with the DNCB-treated group. Similarly, mRNA expression of iNOS decreased (7.4-fold) in the AD+CA-200 group when compared with the AD group. Interestingly, the iNOS mRNA expression in the AD+CA-200 group was slightly reduced when compared with the AD+CA-80 group (Figure 8g). In addition, the expression levels of COX-2 and C-X-C motif chemokine ligand 9 (CXCL9) genes in the AD groups were significantly upregulated in contrast with the CON group (*p* < 0.05) (Figure 8h,i). Expression levels of iNOS, COX-2, and CXCL9 exhibited no statistical differences between the CA treatment group (AD+CA-80 and AD+CA-200) and the AD+Der group (Figure 8g–i). Our results show that CA treatment helps decrease the mRNA expression of inflammatory factors (i.e., iNOS, COX-2, and CXCL9).

### 3.8. Effect of CA on the Protein Expression of Various Pathogenic Cytokines

**Figure 9:**
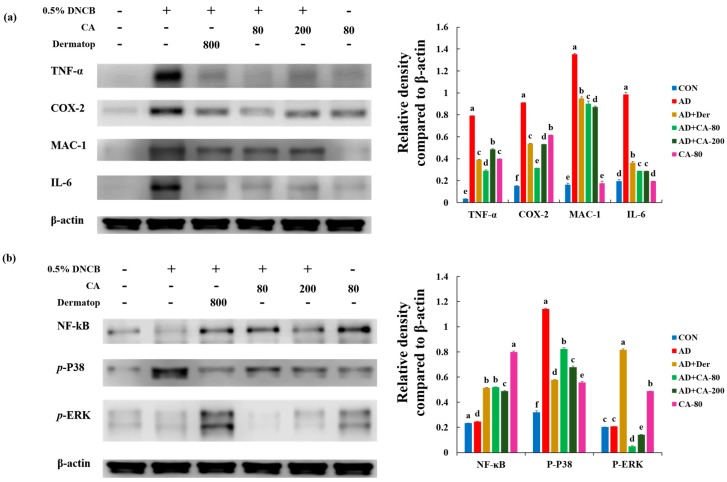
Alleviation of increased expression of (**a**) various pathogenic cytokines and (**b**) NF-κB and mitogen-activated protein kinase (MAPK) proteins in AD-induced mice model, following treatment with CA-80 and CA-200. Values represent the mean ± SD. Values with different letters were significantly different according to Duncan’s multiple range test (*p* < 0.05).

To investigate the anti-inflammatory effects of CA, we examined whether CA would affect the activation of TNF-α, COX-2, MAC-1, and IL-6. As shown in Figure 9a, the expression level of TNF-α in the AD group was significantly higher (22.9-fold) than the CON group; however, this elevation was significantly reduced by treatment with AD+CA-80 and AD+CA-200 (2.7- and 1.6-fold, respectively). The expression of TNF-α decreased 0.7-fold in the AD+CA-80 group as compared with the positive control. The expression of COX-2 increased 5.9-fold in the AD group as compared with the CON group; this enhanced secretion was repressed by treatment with AD+CA-80 and AD+CA-200 (2.9-, and 1.7-fold respectively). Moreover, the AD+CA-80 group significantly decreased (*p* < 0.05) the expression of COX-2 compared with the positive control (1.7-fold). The level of MAC-1 and IL-6 in the AD group were significantly elevated (8.2- and 5.0-fold, respectively) compared with the expression in the CON group, but the MAC-1 and IL-6 level were significantly reduced (*p* < 0.05) after treatment with AD+CA-80 (1.5- and 3.4- fold, respectively). In addition, MAC-1 and IL-6 significantly decreased (1.6- and 3.4-fold, respectively) in the AD+CA-200 group compared with the AD group (*p* < 0.05). In the CA-treated groups, MAC-1 and IL-6 expression were further reduced compared with the positive control. Therefore, these results showed that the CA treatment decreased AD inflammation in DNCB-induced mice.

### 3.9. Effects of CA on NF-κB Modulation and Mitogen-activated protein kinase (MAPK) Signaling Pathways

We assessed the effects of CA on NF-κB and mitogen-activated protein kinases (MAPKs) activation. We measured p-p38, p-ERK, and NF-κB activation in response to CA in ear tissue (Figure 9b). The expression level of p-p38 in the AD group was significantly higher (3.6-fold, *p* < 0.05) than in the CON group. In contrast, the expression level of p-p38 in CA-treated group (AD+CA-80 and AD+CA-200) was suppressed compared to the AD group (1.4- and 1.7-fold, respectively).

Differences between the NF-κB expression levels of AD and CON groups were not statistically significant. Rather, the NF-κB expression increased in the Dermatop and CA treatment groups (AD+Der, AD+CA-80, and AD+CA-200) compared with the AD group (2.0-, 2.1-, and 1.9-fold, respectively). Results of NF-κB expression, in western blotting analysis, was dissimilar to NF-κB data of immunohistochemistry. The expression of p-ERK in the AD group was similar to that observed in the CON group. Both AD+CA-80 and AD+CA-200 treatment decreased p-ERK expression by 4.3- and 1.5-fold when compared with the group only treated with AD. Our results indicate that CA treatment reduces p-p38 expression in the AD model.

## 4. Discussion

For the first time, this study demonstrated the pharmacological effects of CA on DNCB-induced skin inflammation using in vitro and in vivo models. Additionally, our results revealed that CA inhibited IL-6, COX-2, and TNF-α in the DNCB-induced AD mouse model, and that CA inhibits IFN-γ and TNF-α-induced pro-inflammatory mediators, including IL-6 and COX-2 in HaCaT cells. Therefore, CA inhibits the expression of inflammatory cytokines, which can effectively suppress AD symptoms such as the raise epidermal and dermal thickness and infiltration of mast cells and eosinophils into the dermis.

In this study, flavonoid levels in the CA were observed with a peak of 462.42 mg/100 g. Our study suggests that the flavonoid level, observed in CA, contributed to the antioxidant activity of the CA [33]. Moreover, antioxidant activity in *Centella asiatica* depends on the ratio of MO, AO, MA, and AA [31]. Shukla et al. [34] have reported that the improvement of antioxidant activity potentially owes to both AO and flavonoid content in *Centella asiatica* [35]. *Centella asiatica* extract has been reported to possess free radical scavenging activity in ABTS, DPPH, nitric oxide radical scavenging, and oxygen radical absorbance capacity assays in previous reports [31]. Our findings also agree with those of previous studies [31,33,34,35]. Therefore, the potent antioxidant activity of CA is justified by the contents of polyphenol, flavonoid, and the following four triterpenes: MO, AO, MA, and AA.

Mast cells release several crucial signaling molecules, among which histamine has especially potent pro-inflammatory activities [36]. In the present study, CA alleviated the typical and histological phenomena of AD, such as thickening of ear, epidermis and dermis, and increase of mast cell infiltration. Skin treatment and oral administration of CA significantly decreased the typical histopathological changes of AD, and the number of mast cells infiltrating the skin lesions in AD-induced mice.

In previous studies, AD was considered to result from a Th1 and Th2 imbalance [37]. In AD patients, Th2-mediated responses are more prominent in the acute phase, whereas Th1-mediated responses are more prominent in chronic AD disease [38]. Th2 cells mainly secrete IL-4 and IL-5, and these cytokines stimulate B cells, which, in turn, secrete IgE. AD patients are characterized by a surplus of IgE, which, in turn, affects IgG_1_ and IgG_2a_ levels in mast cells, blood, activated by various cytokines and IgE, produce inflammatory cytokines, such as TNF-α, IL-1β, and IL-4 [39].

Th1-mediated inflammation combats intracellular infections through its main cytokine, IFN γ, whereas Th2-associated cytokines such as IL-4 and IL-5 are involved in the fight against extracellular pathogens and allergic responses, and mediate functions such as IgE class switching [37,40]. The associated Th2 inflammatory cytokines IL-4 and IL-6 could promote the outbreak and development of inflammatory reactions [41,42]. In our study, CA application decreased the skin levels of IL-4, IL-5, IL-6, and IL-10 that are induced by DNCB treatment. Initially, CA was evaluated for its cytotoxicity in HaCaT cells. The MTT assay results suggested that CA showed no significant cytotoxicity at the maximum tested concentration (500 µg/mL). During the AD allergy, mast cells produce proinflammatory mediators, such as cytokines and chemokines, upon activation with antigens [28,43]. TNF-α is one of the well-known proinflammatory cytokines and its inhibition is considered a viable strategy for preventing the allergy [44]. Our results indicate that CA provides anti-allergic effects by inhibiting the TNF-α induced from mast cells.

It is reported that over expression of Th2 cytokines, including IL-4, IL-5, and IL-10, are predominant in the acute phase of AD lesions, which stimulate the high production of IgE [45]. Meanwhile, Th1 producing IFN-γ and other proinflammatory cytokines, such as IL-6 and TNF-α, were abundant in the chronic phase of AD [46]. Treatment of CA showed significant potency in suppressing the mRNA expressions of Th1 and Th2 cytokines in both acute and chronic AD-lesion inflammation. Thus, in the ear tissue, expression levels of TNF-α, IL-4, IL-5, IL-6, IL-10, iNOS, COX-2, CXCL9, and MAC-1 were significantly inhibited by CA. The COX-2 enzyme, one of the isoforms of COX, plays prominent roles in allergic reactions and mast cell-mediated inflammations [27,47]. As inflammatory cytokines and endotoxins induce COX-2 enzymes, alleviating upregulated COX-2 and inflammatory mediator expression can be considered as an attractive strategy for the development of anti-allergic drugs [48]. CA showed considerable effects in suppressing protein expression level, including TGF-β, TNF-α, iNOS, and COX-2. Thus, CA can be an anti-allergic drug model by deterring mast cell-dependent inflammatory mediators.

Investigation of CA on AD-like skin lesion in the BALB/c mouse model revealed that the major components of CA, such as MO and AO, not only have significant potential on reducing the thickness on the dorsal skin surface, but also on reducing the thickness of epidermal and dermal thickness, proving their impact on skin hypertrophy and granulocyte infiltration. Usually, in IgE-mediated allergies including AD, elevated levels of mast cells are predominant [49]. Upon activation of mast cells, histamine is released as one of the immune mediators; thus, elevated levels of histamine have been observed in AD patients [50]. The suppression effect on mast cell infiltration exerted by MO and AO suggest that they can be considered as a possible model for designing drugs for IgE mediated allergies.

MAPK signaling is indispensable in the pathogenesis of the inflammatory response [51,52]. Topical p38 MAPK inhibition mitigates severe inflammatory responses in the dermis [53]. Proinflammatory factors, including TNF-α and IL-6, bind their receptors to promote a phosphorylation cascade that ultimately activates p38 and JNK to induce more cytokine production, resulting in an amplifying loop [54,55]. Our result suggests that CA effectively reduces inflammation and balances the immune response by inhibiting p38 signaling. However, further clinical studies require confirming the anti-atopic dermatitis effects of CA on human skin.

## 5. Conclusions

In conclusion, the findings of this study demonstrate that skin local and oral administration of CA ameliorated DNCB-induced AD-like skin inflammation, which may be due to reduced infiltration of immune cells and subsequent Th1 and Th2 responses in AD skin and decreased IFN-γ/TNF-α-promoted proinflammatory cytokines and chemokines in keratinocytes. In addition, the CA showed a substantial effect in suppressing histopathological changes in mice, including cytokine expression and protein levels of TNF-a, COX-2, MAC-1, and IL-6. The main components of CA were MO and AO. The immunosuppressive effect of CA may be due to the synergism of particular bioactive compounds in possession of potential anti-allergic activities and anti-inflammatory. Taken together, our data suggest that CA could potentially be used as a useful pharmacological agent.

## Supplementary Materials

The following are available online at https://www.mdpi.com/2072-6643/12/2/411/s1, Table S1: List of primers for real-time PCR.
