# Supplementary material for: Inhibitory Effect of Centella asiatica Extract on DNCB-Induced Atopic Dermatitis in HaCaT Cells and BALB/c Mice
Source: Nutrients. 2020 Feb 5;12(2):411. doi: 10.3390/nu12020411 (PMC7071208; doi:10.3390/nu12020411)
Supplement: Supplementary file 1 [file nutrients-12-00411-s001.pdf]

SUPPLEMENTARY FILE

Table 1. List of primers for real-time PCR.

Name	Forward	Revers
GAPDH	5′-CATGGCCTTCCGTGTTCCTA-3′	5′-TGTCATCATACTTGGCAGGTTTCT-3′
TNF-α	5′-AAGCCTGTAGCCCACGTCGTA-3′	5′-GGCACCACTAGTTGGTTGTCTTTG-3′
IL-4	5′-TCTCGAATGTACCAGGAGCCATATC-3′	5′-AGCACCTTGGAAGCCCTACAGA-3′
IL-5	5′-ACAGGAGAAGGGACGCCAT-3′	5′-GAAGCCGTACAGACGAGCTCA-3′
IL-17	5′-TCCCCTCTGTCATCTGGGAAG-3′	5′-CTCGACCCTGAAAGTGAAGG-3′
IL-6	5′-CCACTTCACAAGTCGGAGGCTTA-3′	5′-GCAAGTGCATCATCGTTGTTCATAC-3′
IL-10	5′-TCAGCTGTGTCTGGGCCACT-3′	5′-TTATGAGTAGGGACAGGAAGCCTCA-3′
iNOS	5′-GGAATGGAGACTGTCCCAGCA-3′	5′-GTCATGAGCAAAGGCGCAGA-3′
COX-2	5′-GCCAGGCTGAACTTCGAAACA-3′	5′-GCTCACGAGGCCACTGATACCTA-3′
CXCL9	5′-GGAACCCTAGTGATAAGGAATGCA-3′	5′-TGTCATCATACTTGGCAGGTTTCT-3′
